# The Prognostic Role of Cuproptosis in Head and Neck Squamous Cell Carcinoma Patients: A Comprehensive Analysis

**DOI:** 10.1155/2022/9996946

**Published:** 2022-09-02

**Authors:** Qin Ding, Xiaochuan Chen, Wenquan Hong, Lihua Wang, Wei Liu, Sunqin Cai, Xin Chen, Jun Lu, Sufang Qiu

**Affiliations:** ^1^Clinical Oncology School of Fujian Medical University, Fujian Cancer Hospital, Fuzhou, China; ^2^Fujian Provincial Key Laboratory of Translational Cancer Medicine, Fuzhou, China; ^3^Department of Radiation Oncology, Clinical Oncology School of Fujian Medical University, Fujian Cancer Hospital, Fuzhou, China

## Abstract

**Purpose:**

Head and neck squamous cell carcinoma (HNSCC) exhibits a high mortality and morbidity rate, and its treatment is facing clinical challenges. Cuproptosis, a copper-dependent cell death process, can help derive new forms of cancer therapies. However, the potential of cuproptosis-related genes (CRGs) as novel biomarkers for risk prediction, screening, and prognosis remains to be further explained in HNSCC.

**Methods:**

We built a prognostic multigene signature with CRGs, which is associated with the tumor immune microenvironment (TME) by gene set enrichment analysis (GSEA), in the TCGA cohort. Furthermore, we systematically correlated risk signature with immunological characteristics in TME including tumor-infiltrating immune cells (TIICs), immune checkpoints, T cell inflamed score, and cancer immunity cycles. We also thoroughly investigated the biological functions of cuproptosis-associated lncRNAs and its immunological characteristics.

**Results:**

CRGs-related prognostic model showed good prediction performance. A higher risk score was associated with a poorer overall survival (OS) than those with low-risk scores, according to the results of the survival analysis (*p* < 0.0001). The risk score was significantly related to the variable clinicopathological factors. Samples with high-risk scores had lower levels of CD8+ T cells infiltration. Immune therapy might be effective for the low-risk subtype of HNSCC patients (*p* < 0.05). Moreover, 11 differentially expressed lncRNAs as the independent prognostic factor could also predict TME in an accurate manner.

**Conclusion:**

Our study identified and validated novel cuproptosis-related biomarkers for HNSCC prognosis and screening, which offer better insights into developing accurate, reliable, and novel cancer therapies in the era of precision medicine.

## 1. Introduction

Head and neck squamous cell carcinoma (HNSCC) is recorded to be the 8th most commonly occurring form of cancer in the world, with a high mortality and morbidity rate [1, 2]. Approximately 600,000 new HNSCC cases and 350,000 HNSCC-related deaths take place every year across the globe [3–5]. Though many new techniques have been developed for diagnosing the patients during their early stages, more than half of the HNSSC patients are diagnosed only in their advanced stages [6]. In the past few years, many significant breakthroughs have been achieved in the HNSCC treatment, including chemotherapy, radiotherapy, and surgery. Despite these breakthroughs, the 5-year survival rate of HNSCC patients is still poor [7, 8]. Hence, it is essential to consider alternative probable causes of cell death to overcome the chemotherapy resistance and identify novel biomarkers that can be used as therapeutic agents for improving the prognosis of patients with HNSCC.

Many researchers are investigating the role of cuproptosis in cancer. Protein lipoylation is mainly responsible for copper-induced cell death (i.e., cuproptosis). In their study, Tsvetkov et al. identified a new type of copper-induced cell death mechanism, where the Cu ions disrupted some particular mitochondrial metabolic enzymes, which was more toxic in the actively respiring cells [9, 10]. An increase in the number of respiratory cells increases the level of the lipoylated enzymes, which, in turn, leads to the formation of more protein aggregates [10, 11]. It is concluded that copper ions can sabotage the cell and cause the death of cells if the metabolic pathway in the cells was disrupted [12, 13]. Hence, we believe that an investigation into the copper toxicity-based pathology can help determine and uncover the genetic diseases related to genetic copper overload and derive new forms of cancer therapies.

Very few researchers have investigated the role played by the cuproptosis-related molecular signatures in predicting the overall survival (OS) rate of HNSCC patients. In this study, we have evaluated the expression profile of cuproptosis-related genes (CRGs), initially described by Tsvetkov et al. [10], and developed a prognostic multigene signature that was based on the CRGs. Additionally, we also studied the role of the CRGs, immune tumor microenvironment, lncRNAs, and immune responses in HNSCC.

## 2. Materials and Methods

### 2.1. Data Collection

In this study, we downloaded the dataset that included the mRNA expression and the related clinical data for 502 tumor tissues along with 44 adjacent normal tissues of HNSCC, from The Cancer Genome Atlas (TCGA; https://portal.gdc.cancer.gov/) (Table 1).

Thereafter, we retrieved 10 CRGs (Table 2) from the published studies [10]. We used the Clinical Proteomic Tumor Analysis Consortium (CPTAC, https://proteomics.cancer.gov/programs/cptac), developed by the National Cancer Institute, and a cBioPortal (http://www.cbioportal.org/) for the purpose of comparing the methylation sequences, RNAseq, and proteomics data between the tumor and nontumor tissues. The correlation between the CRGs and lncRNAs was determined with the help of the Pearson correlation analysis. Based on the values of the correlation coefficient, |*R*^2^| > 0.1, and *p* < 0.05, the cuproptosis-associated lncRNAs were regarded as statistically significant lncRNAs. Furthermore, we conducted the functional analysis with the help of the Kyoto Encyclopedia of Genes and Genomes (KEGG) and Gene Ontology (GO) databases, by implementing the R language ggplot2 package for assessing the roles of all identified CRGs.

### 2.2. Designing the Cuproptosis-Associated Prognostic Gene Signature

We used the Lasso-penalized Cox regression technique for developing the cuproptosis-associated prognostic gene and lncRNA signatures. We used the formula described below for predicting the risk score of every HNSCC patient:

(1) $$\begin{array}{c} \text{risk score}=\overset{\text{n}}{\underset{i=1}{\sum }}\left(\exp\times \text{coef}\right), \end{array}$$

where exp denotes the gene and the lncRNA expression value, while *coef* refers to the coefficient of a gene and lncRNA in LASSO analysis. We also downloaded and studied the related clinical data of HNSSC patients. We classified this data as low-risk (with a value lesser than the median number) or high-risk (with a value higher than the median number) groups. We used the Kaplan-Meier survival analysis for assessing the survival rate. Then, we tested these two signatures with the help of the univariate and multivariate Cox regression models for determining if they were independent prognostic factors or not. Finally, we used the receiver-operator characteristics (ROC) and the respective areas under the curve (AUC) for analyzing the performance of the prediction models.

### 2.3. Assessing the Immunological Characteristics of TME

We estimated the stromal, immune, and ESTIMATE scores using the R package “ESTIMATE.” Immunological features of the TME include the inhibitory immune checkpoints, immunomodulators, and tumor-infiltrating immune cells (TIICs). We acquired the data regarding the 92 immunomodulators such as receptors, chemokines, and MHC from an earlier study [14]. We used the MCP-counter, Cibersort, Cibersort-ABS, quanTIseq, xCell, TIMER, and EPIC algorithms for assessing the TIIC infiltration level in TME [15–19]. We also derived the effector genes of the TIICs from multiple earlier studies [20].

From an earlier study, we retrieved a group of 10 inhibitory immune checkpoints that displayed therapeutic efficacy [21]. A gene set that showed a T cell-inflamed gene expression profile (GEP) and included 18 inflammatory genes was downloaded from an earlier study (Table 3) [22]. We also derived the Shannon Entropy data of the T cell receptor (TCR) and B cell receptor (BCR) from an earlier report [23].

We acquired the Microsatellite Instability (MSI) for somatic mutation analysis from an earlier report [24]. We assessed the tumor mutation burden (TMB) and the mutant-allele tumor heterogeneity (MATH) data, which comprised the wound healing, silent and non-silent mutation rate, lymphocyte infiltration signature score, fraction altered, and macrophage regulation from the somatic mutation data for 465 tumor samples, with the help of the “maftools” R package [24, 25].

The anticarcinoma immune response was reflected in the cancer immunity cycle using 7 steps (Table 4). The fate of tumor cells was determined throughout the procedure. Thereafter, we used a single-sample gene set enrichment analysis (ssGSEA) technique for assessing the gene expression of single samples [19].

### 2.4. Prediction of the Response of Comprehensive Therapy

By constructing the ridge regression model based on Genomics of Drug Sensitivity in Cancer (GDSC) (http://www.cancerrxgene.org/) cell line expression spectrum and TCGA gene expression profiles, the “pRRophetic” package in R could be applied to predict the half-maximal inhibitory concentration (IC50) of chemotherapy in the high- and low-risk groups of HNSCC patients and to infer the sensitivity of the different patients [26].

### 2.5. Statistical Analysis

All statistical analyses were done on R version 3.6.0. Continuous variables were compared between the two groups using Wilcoxon rank sum test. Categorical variables were compared between the groups using the chi-square test. The prognostic value of categorical variables was assessed using the log-rank test. For all analyses, two-paired *p* value ≤ 0.05 indicated statistically significant differences. ∗, ∗∗, ∗∗∗, and ∗∗∗∗ indicate *p* value ≤ 0.05, <0.01, <0.001, and<0.0001, respectively.

## 3. Results

### 3.1. Identification of CRGs and Expression Profile in HNSCC

**Figure 1:**
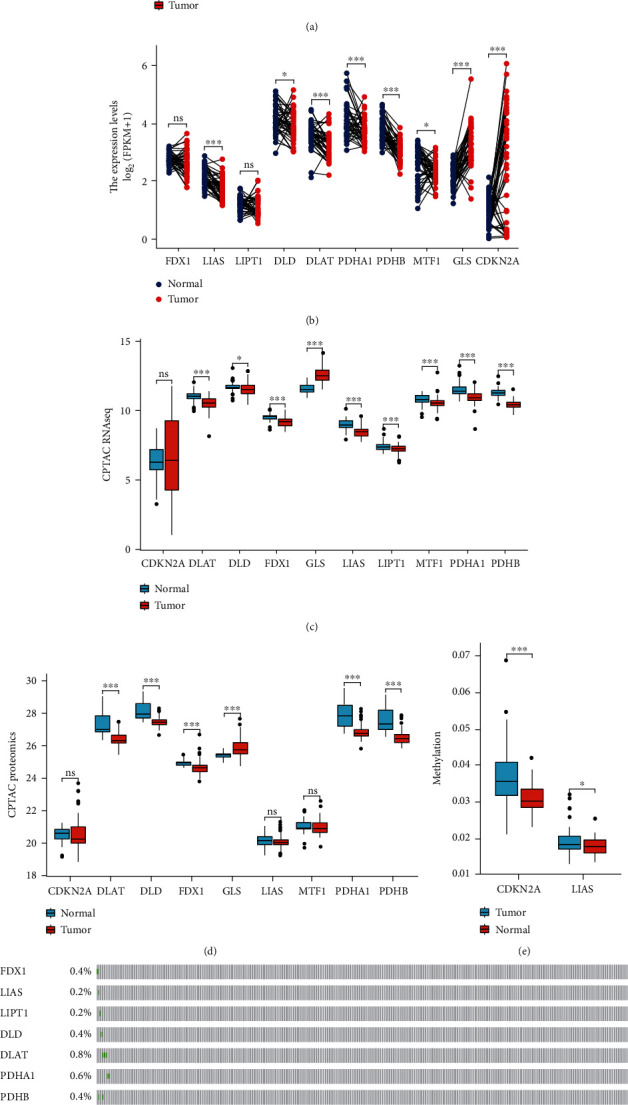
Expression profile of cuproptosis-related genes. (a), (b) Box plots present the differentially expressed cuproptosis-related genes between HNSCC and normal sample. (c), (d) Box plots validated the expression profile from CPTAC RNAseq and proteomics data. (e) The methylation degree of CDKN2A and LIAS. (f) Mutation status.

The results showed that a majority of CRGs (8/10, 80%) were expressed differentially in the tumor and adjacent nontumor tissues (Figures 1(a) and 1(b)), which was validated (9/10, 90%) with the help of the CPTAP RNAseq (Figure 1(c)) and CPTAC Proteomics data (Figure 1(d)). Moreover, differential CDKN2A and LIAS methylation levels were noted in the tumor and adjacent nontumor tissues (Figure 1(e)). Next, we studied the genetic mutations in the CRGs and identified CDKN2A as the gene that underwent the most frequent mutations (Figure 1(f)).

### 3.2. Development of a Prognostic Cuproptosis-Related Gene Model

**Figure 2:**
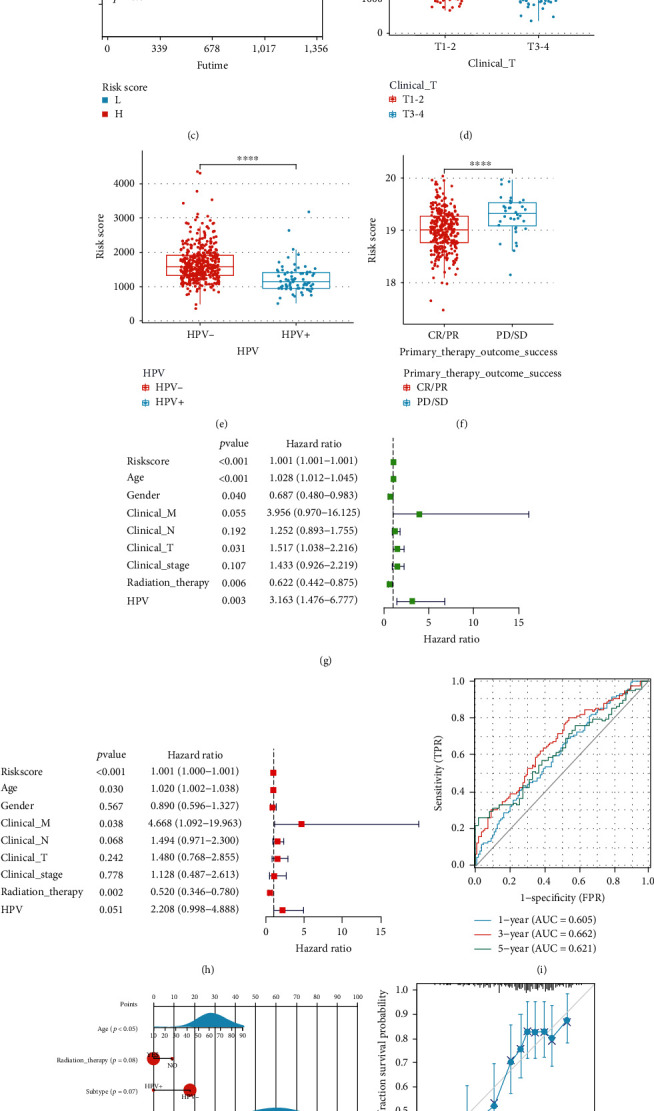
Construction of CRGs prognostic model and survival analysis. (a) The 7 CRGs used for construction of the gene risk model. (b), (c) In TCGA and CPTAC cohorts, low-risk group patients had favorable OS rates compared with those in the high-risk group. (d–f) Correlation analysis of risk score and clinical characteristics. (g), (h) Cox analyses, univariate and multivariate, showing the independent prognostic significance of the risk signature in predicting the OS of HNSCC patients in the TCGA cohort. (i) The ROC curve for 1-, 3-, and 5-year OS of HNSCC patients in the TCGA cohorts. (j) The predicted the 1-year, 3-year, and 5-year OS of HNSCC patients based on the constructed nomogram. (k), (l) Calibration curve of the OS predicted by the nomogram model. The dashed diagonal line represents the ideal nomogram. (m) DCA curve for 1- and 3-year prognosis.

We implemented the LASSO Cox regression analysis for developing a prognostic model that was based on the expression profile of the 10 abovementioned genes. They identified a 7-gene signature based on the optimal *λ* value (Figure 2(a)). Table 2 presents a list of the coefficients of every gene.

We carried out the survival analyses using the values of the risk score and observed that a higher risk score was associated with a poor prognosis (*p* < 0.05, Figures 2(b), S1(a), and S1(b)), which was further validated using the CPTAC clinical data (Figure 2(c)). It has been reported the expression of CRGs may be correlated with disease grade in clear renal cell carcinoma, hepatocellular carcinoma, and melanoma [27–29]. In our study, the high-risk group was significantly related to a higher clinical T stage (Figure 2(d)), HPV^−^(Figure 2(e)), PD/SD (Figure 2(f)), higher pathologic T stage (Figure S1(c)), a higher number of positive lymph nodes (Figure S1(d)), and higher grade and shorter PFS (Figure S1(e)) in the TCGA cohort. We determined the risk factors for establishing a 7-CRG-based prognostic model. Our results confirmed that the age, risk score, and radiation therapy could be considered independent prognostic factors for OS (Figures 2(g) and 2(h)). We used the molecular signature for predicting the AUC values of the 1-, 3-, and 5-year survival rates of the patients to be 0.605, 0.662, and 0.621, respectively (Figure 2(i)). Thereafter, we combined the prognostic and clinical pathology models for constructing a nomogram (Figure 2(j)). This combination improved the predictive value of OS over 1, 3, and 5 years and can be effectively used for informing the clinical management about the ideal predictive performance (Figures 2(k) and 2(l)) and clinical advantages (Figure 2(m)). Furthermore, we used a heat map library for determining the risk scores. While assessing the predictive ability of the risk scores, we classified the patients into the low-risk and high-risk groups. We presented the gene heat maps and population follow-up time in order of the ranking (Figure S1(f)). It was noted that the survival rate of the patients decreased with an increase in the risk score.

### 3.3. Functional Analyses in the TCGA Cohort

**Figure 3:**
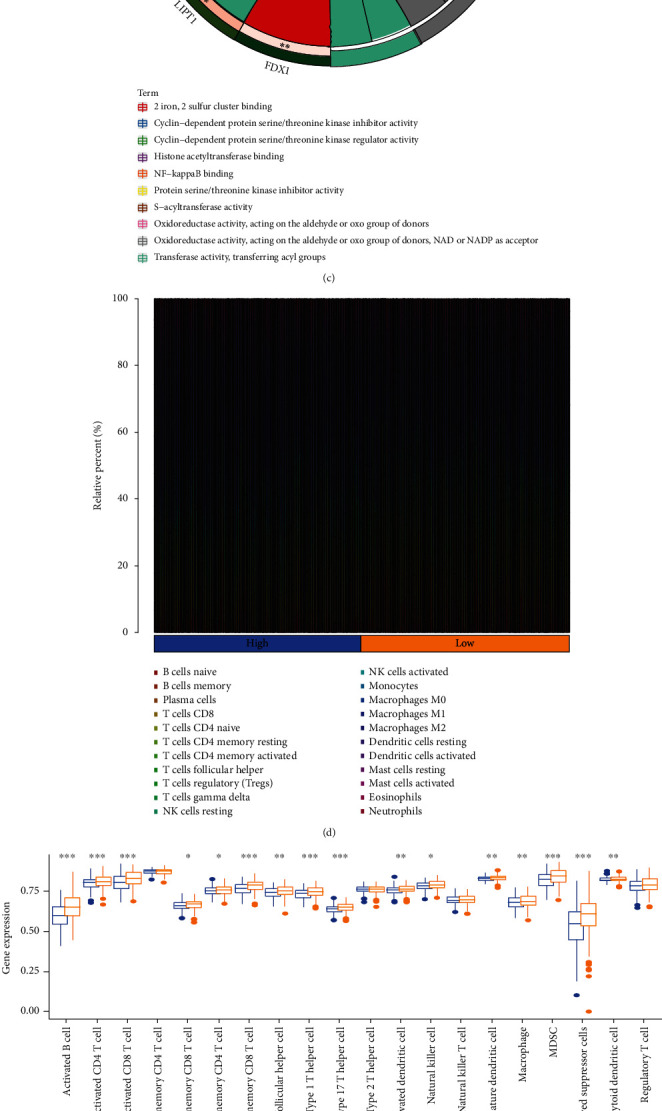
Correlation between the risk signature and immune microenvironment of HNSCC. (a)–(c) Enrichment plots from gene set enrichment analysis (GSEA). (d) Relative proportion of immune infiltration in HNSCC patients. (e) Box plot shows the differential immune infiltration between low-risk and high-risk groups. (f) Box plot present the differential stromal score, immune score, and ESTIMATE score between low-risk and high-risk groups. (g) Relationship between the risk score and infiltration levels of severe TIICs, as determined by seven separate algorithms.

After the above steps, we carried out the functional annotation using GSEA and identified 5 enriched KEGG pathways. We noted that the intestinal immune network for T cell receptor signaling pathway, Fc epsilon RI signaling pathway, IgA production, B cell receptor signaling pathway, and the primary immunodeficiency pathways were subjected to enrichment in the low-risk group (Figure 3(a)). Additionally, the Gene Ontology (GO) terms such as immune response regulating cell surface receptor signaling pathway, cell recognition, B cell-mediated immunity, Fc epsilon receptor signaling pathway, Fc receptor signaling pathway, Fc receptor-mediated stimulatory signaling pathway, and the humoral immune response were enriched in the HNSC samples and exhibited a low-risk score (Figure 3(b)).  Figure 3(c) presents the 5 CRGs that were enriched in the cancer-related pathway, such as the NF-*κ*B pathway. For determining the relationship between the risk scores and immune status, we determined the enrichment scores of various immune cell sub-populations using ssGSEA. We assessed the immune landscape of 22 immune cell types in the HNSCC patients with the help of the ssGSEA technique (Figure 3(d)). Comparing the data in the TCGA cohort highlighted the varying number of activated CD4 T cells, activated B cells, activated CD8 T cells, follicular helper CD8 T cells, effector memory CD8 T cells, and natural killer cells in the two risk groups (*p* < 0.05, Figure 3(e)). This study showed that the ESTIMATE and immune scores were inversely linked to the risk scores (Figure 3(f)). For avoiding any errors in the calculations, we estimated the infiltration level of the TIICs using 7 algorithms, i.e., MCP-counter, Cibersort, Cibersort-ABS, TIMER, xCell, quanTIseq, and EPIC (Figure 3(g)).

### 3.4. Prediction of the Tumor Immune Microenvironment Using the Risk Model

**Figure 4:**
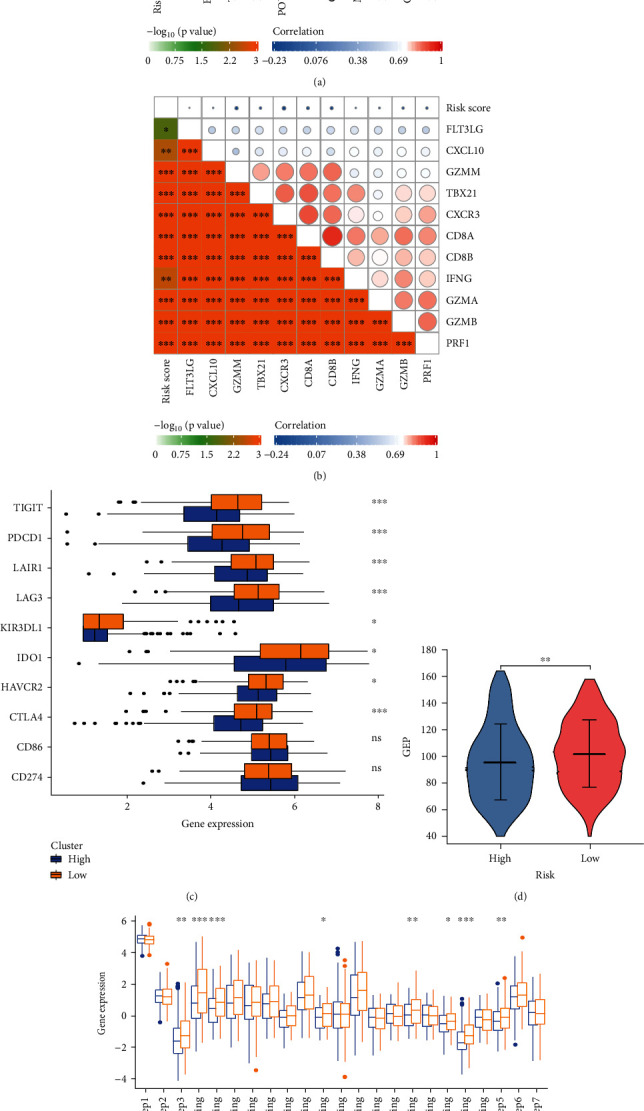
Immune-related functions and in high- and low-risk groups. (a) Relationship between risk score and B cell effector genes. (b) Relationship between risk score and T cell effector genes. (c) Relationship between risk score and 10 inhibitory immune checkpoints. (d) Differences in GEP (T cell-inflamed gene expression profile) between low- and high-risk groups. (e) Difference between low- and high-risk groups at distinct stages of the cancer immunity cycle.

Here, we noted that the risk signature was inversely related to the B cell and CD8+ T cells effector genes (Figures 4(a) and 4(b)). This risk signature was negatively related to a majority of the immune checkpoint inhibitors, like TIGIT, LAIR, PDCD1, LAG3, KIR3DL1, HAVCR2, IDO1, and CTLA-4 (Figure 4(c)). Additionally, the risk signature exhibited a strong correlation with the T cell-inflamed GEP in the HNSCC, which further showed an increase in the low-risk score group (Figure 4(d)). The anticancer immune response is reflected in the cancer immunity cycle consisting of seven phases [14]. A majority of the components in the immune cycle, like the priming and activation (Step 3), as well as immune cell transportation to the tumors (Step 4) (recruiting monocytes, CD4 T cells, Th2 cells, Th17 cells, and Tregs), were found to be higher in the low-risk score group (Figure 4(e)).

**Figure 5:**
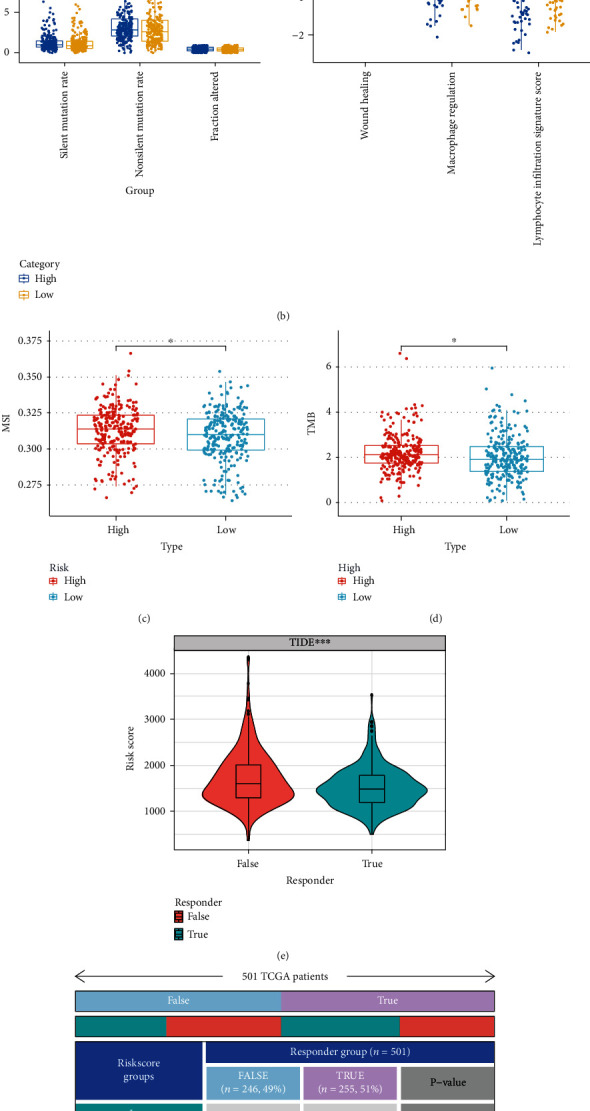
Correlation of the risk score with immune scores, immunotherapy biomarkers, and conventional therapy. (a) Differences in TCR and BCR diversity values between low- and high-risk groups. (b) Differences in MATH (including silent mutation rate, non-silent mutation rate, fraction altered, wound healing macrophage regulation, and lymphocyte infiltration signature score) between low- and high-risk groups. (c)–(d) Differences in MSI and TMB between low- and high-risk groups. (e) Differences in TIDE score between low- and high-risk groups in the TCGA cohort. (f) The anticipated immunotherapy (TRUE/FALSE) response rate to anti-PD-L1 in low- and high-risk groups in the TCGA cohort. (g)–(h) The IC50 of cisplatin and gemcitabine in the low-risk group were higher than those in the high-risk group.

The abilities of the TCR and BCR from the TCGA high-grade serous ovarian cancer (HGSOC) cohort were then examined. The mean TCR and BCR diversity values were variable based on the risk signature, wherein the low-risk score group showed the maximal diversity (Figure 5(a)). Then, we investigated MATH, TMB, and MSI data and observed that the patients in the high-risk score group showed a higher MATH score (which included the silent mutation rate, non-silent mutation rate, wound healing, macrophage regulation, fraction altered, and the lymphocyte infiltration signature score), TMB score, and MSI score (Figures 5(b)–5(d)). We used the TIDE algorithm for predicting the immune checkpoint blockade (ICB) responses to help identify patients who might benefit from immunotherapy. Compared to the patients in the high-risk score group, patients belonging to the low-risk group showed a significantly better response to immunotherapy (Figures 5(e)–5(f)). Furthermore, we estimated the IC50 for every subtype using the predictive model of gemcitabine, cisplatin, doxorubicin, and docetaxel, similar to the technique proposed by Wang [26]. The results of these experiments indicated that the patients with a high-risk score were more susceptible to chemotherapy than the low-risk patients (gemcitabine: *p* = 9.7E6; cisplatin: *p* = 8.0E − 4; docetaxel: *p* = 6.0E − 5; and doxorubicin, *p* = 3.1E3; (Figures 5(g)–5(h)) and S2(a) and S2(b)).

### 3.5. Identifying the Cuproptosis-Associated lncRNAs Based on their Prognostic Value in TCGA

**Figure 6:**
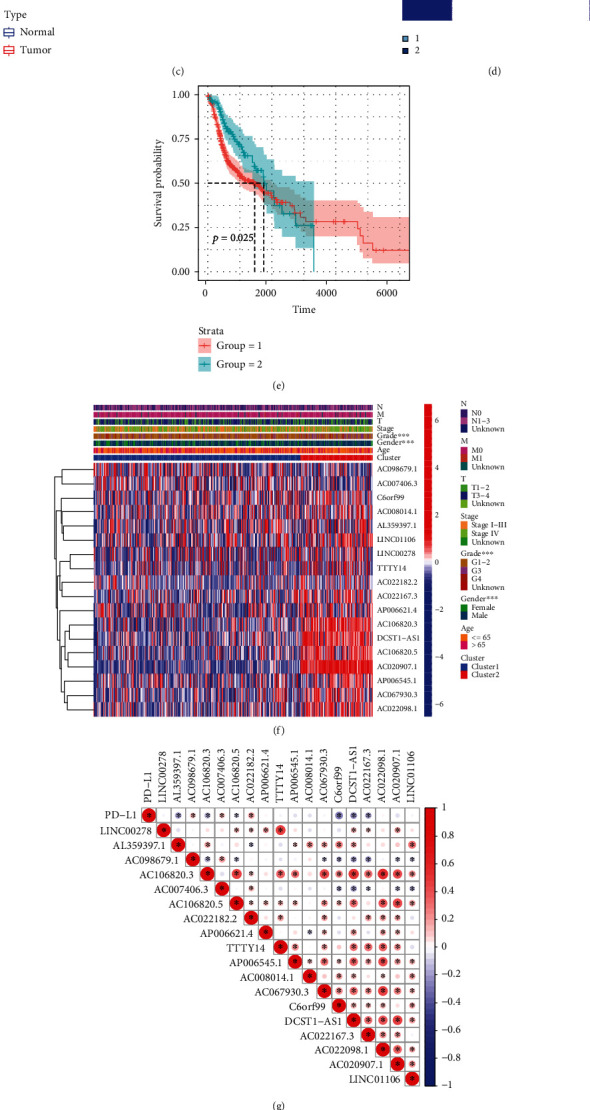
Identification of cuproptosis-associated 18-lncRNAs with prognostic value and immune response in HNSCC patients. (a) A coexpression network of cuproptosis-related lncRNAs and genes was constructed and visualized. (b) 18 independent prognostic predictor lncRNA signatures with different expressions of HNSCC. (c) Box plot presents the differentially expressed cuproptosis-related lncRNAs between HNSCC and normal sample. (d) 501 HNSCC samples from the TCGA cohort were classified into 2 clusters using an unsupervised clustering method. (e) The Kaplan-Meier curve showed that patients in cluster 2 displayed a shorter overall survival than those in cluster 1. (f) Heat map of the prognostic characteristics and clinicopathological correlation of cuproptosis-related lncRNAs. (g) Correlation analysis of PD-L1 expression and 18 independent prognostic predictor lncRNA signatures. (h) Violin plot of immune-infiltrating lymphocytes between cluster 1 and cluster 2. (i) Box plot presents the differentially infiltrated CD8 T cells between cluster 1 and cluster 2. (j) Differences in ESTIMATE score between cluster 1 and cluster 2.

Here, we established the CRGs and lncRNA networks (Figure 6(a)). They identified 109 cuproptosis-related lncRNAs using the Pearson correlation analysis (|cor| > 0.1, Pp < 0.05). Thereafter, they used the univariate Cox regression and identified the 18 lncRNAs which showed an expression level that was associated with the prognosis of the patients, thus demonstrating that they exhibited a prognostic predictive value (*p* < 0.05, Figure 6(b)). The results showed that all cuproptosis-related lncRNAs (11/11, 100%) were expressed differentially between the tumor and adjacent nontumor tissues (Figure 6(c)).

### 3.6. Classification Subtypes Using the Homogeneous Cuproptosis-Related lncRNAs

We used an unsupervised clustering technique for classifying 501 HNSCC samples into two groups from the TCGA cohort (Figure 6(d)). A survival analysis, depending on the subtype, showed that cluster 2 was associated with a poor prognosis (Figure 6(e)).  Figure 6(f) depicts the expression of the 18 lncRNAs, as well as risk scores and clinicopathological variables. In addition, PD-L1 was significantly associated with most of the lncRNAs mentioned above (Figure 6(g)). Patients with TCGA in the cluster 2 had higher ratios of naive B cell (*p* = 0.004), CD8 T cell (*p* < 0.001), and activated resting CD4 T cells (*p* < 0.001) (Figures 6(h)–6(i) and S3(a)–S3(e)). The ESTIMATE and stromal scores were lower in cluster 2 (Figures 6(j) and S3(f)).

### 3.7. Construction of Prognostic Cuproptosis-Related lncRNA Signature

**Figure 7:**
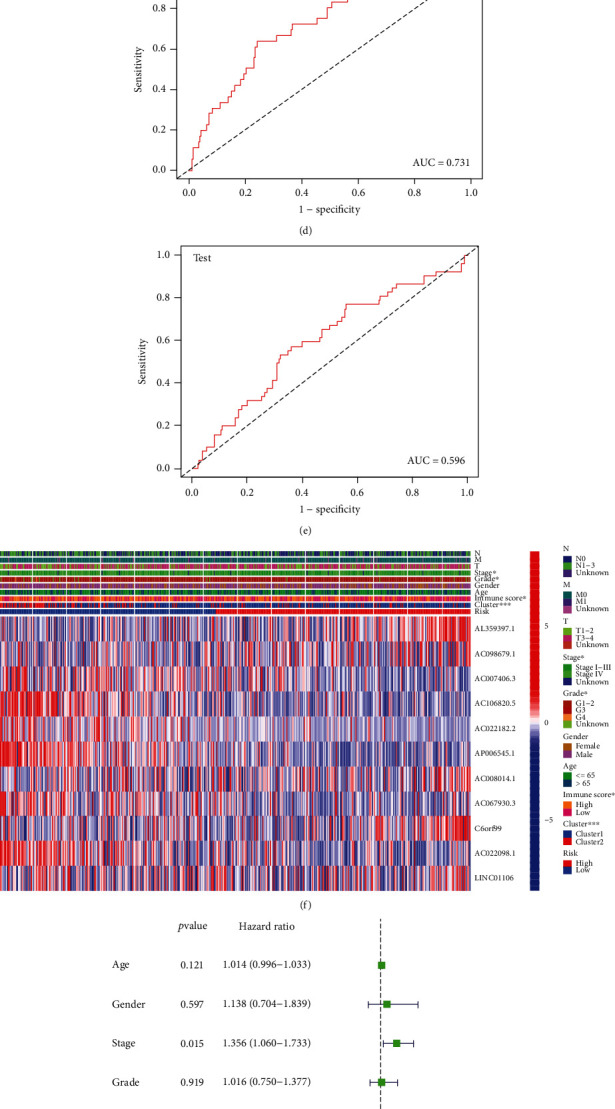
Construction of prognostic cuproptosis-related lncRNA signature. (a) The 11 cuproptosis-related lncRNA used for construction of the gene risk model. (b)–(c) Survival analysis show the prognosis of high-risk and low-risk patients in the train group (b) and the test group (c). (d)–(e) The ROC for risk score with OS for HNSCC cohorts in the train group (d) and the test group (e). (f) Heat map of the prognostic characteristics and clinicopathological correlation based on lncRNA-related risk score. (g)–(j) Univariate and multivariate Cox regression analyses for the lncRNA-related risk score model as an independent prognostic factor both in the train group (g), (h) and the test group (i), (j).

To construct an optimal cuproptosis-related lncRNA risk model for predicting HNSCC prognosis, the 11 cuproptosis-related lncRNA were identified using LASSO regression analysis (Figure 7(a)). Meanwhile, the 501 HNSCC patients were divided into the train group and test group for internal verification. The coefficients of each lncRNA are listed in Table 3.

The results for both the train and the test groups indicated that the low-risk patients experienced a longer OS period in comparison to the high-risk patients (Figures 7(b) and 7(c)). We used the ROC curves for assessing whether the expression profiles of cuproptosis-related lncRNAs could be employed as a potential biomarker for predicting the onset and progression of HNSCC. An AUC of 0.731 was observed in the train group, while the test group showed an AUC of 0.596, implying that this prognostic model is specific and fairly sensitive (Figures 7(d) and 7(e)).

**Figure 8:**
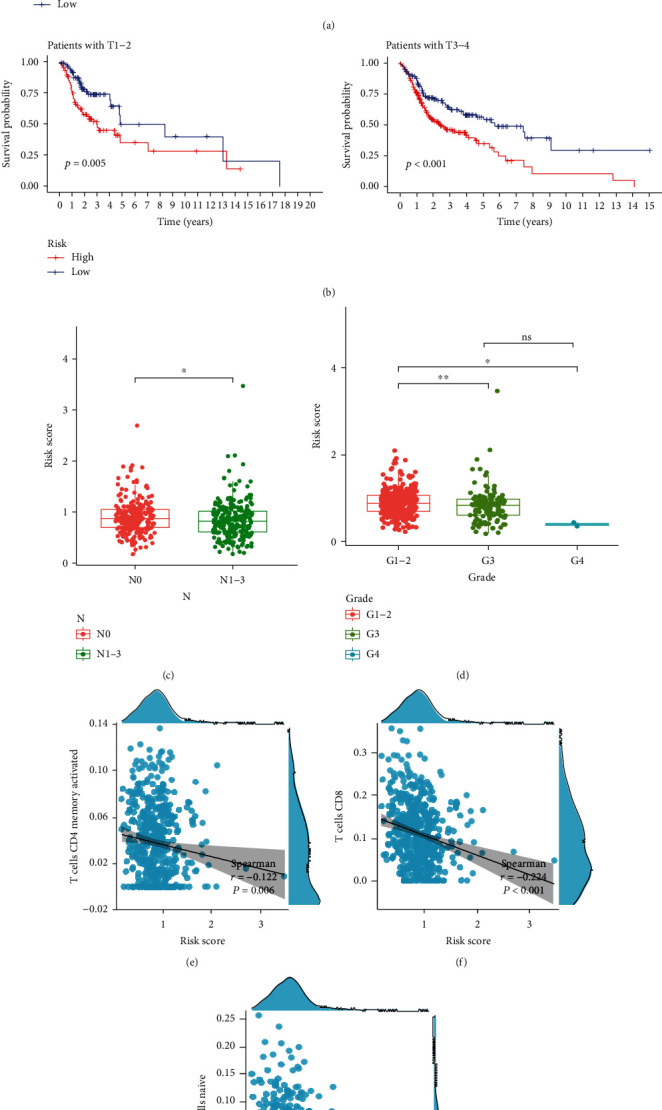
Prognostic value of cuproptosis-associated lncRNA signature and the correlation with relevant immune cells. (a), (b) The Kaplan-Meier curve showed that patients in different groups ((a) patients with different age; (b) patients with T stage) with high risk displayed a shorter overall survival than those with low risk. (c), (d) Correlation analysis of lncRNA-related risk score and clinical characteristics. (e)–(g) lncRNA-related risk score was inversely correlated with CD4 memory activated T cells (e), CD8 T cells (f), and naive B cells (g).

Figure 7(f) depicts the expression of 11 cuproptosis-related lncRNAs, as well as clinicopathological variables, clusters, and risk scores. Then, we carried out the univariate and multivariate Cox regression analyses for determining if the cuproptosis-related lncRNAs could be applied as an independent prognostic model for assessing the OS in HNSCC patients. The results indicated that the cuproptosis-related lncRNA risk model was an effective independent prognostic indicator in the test and the train groups (Figures 7(g)–7(j)). Furthermore, the risk-score values were quantified with the aid of the heat map library, demonstrating that high-risk scores were related to shorter survival times (Figures S3(g)–(h)). According to the survival analysis, the low-risk patients showed a longer survival time than the high-risk patients (Figures 8(a) and 8(b) and S4(a)–(i)). We further observed that the stage, grade, immune scores, and clusters were differently distributed between the high and low-risk groups, which were further demonstrated in Figures 8(c)–8(d) and S5(a)–S5(b). Additionally, we investigated the relationship between the lncRNA risk model and immune cells and observed that the risk model was negatively correlated to the CD8 T cells, CD4 memory-activated T cells, and naive B cells (Figures 8(e)–8(g)). However, it was positively related to the CD4 memory resting T cells (Figure S5(c)).

## 4. Discussion

In the past decade, reports showed that mammalian cells are harmed by essential trace metals. Metals have emerged as a promising new method of killing cells other than via apoptosis. The new mechanism reported by Tsvetkov et al. suggests that the use of copper may be particularly beneficial for cancer patients that are naturally resistant to apoptosis, representing a new method to kill cancer cells as well as offer a potential treatment for tumors [10].

In this study, we investigated the CRG expression profiles and TCGA dataset to determine the cuproptosis-related prognostic gene signatures. A clinical dataset of HNSCC patients from the CPTAC was used for validating the signature. Moreover, we also identified the role of cuproptosis-related genes and constructed a corresponding lncRNAs signature. The study also examined TIICs, TMEs, immune function, immune checkpoints, and immunotherapy as the possible immune responses. Additionally, this study investigated novel therapeutic targets based on the potential and novel biomarkers of the cuproptosis-related pathways in HNSCC.

Recent advances in high throughput DNA sequencing have made it possible to fully characterize the somatic mutations of cancer. As with other cancers associated with solid and smoking-related cancers, HNSCC is formed by the accumulation of a variety of genetic and epigenetic changes [30–32]. Our study demonstrates the mutation of cuproptosis-related genes in HNSCC, with the greatest extent of mutations in CDKN2A, suggesting a possible involvement in the development and progression of HNSCC. A differential expression was noted in eight out of 10 hub CRGs between the tumor and normal tissues, as per the data derived from the TCGA and CPTAC. These results suggested that 10 CRGs were associated with the development of HNSCC.

Then, we validated the accuracy of risk models in predicting the OS of HNSCC patients. The prognostic signature was regarded as a trustworthy technique for anticipating the prognosis of patients with HNSCC and showed satisfactory prognostic discrimination in patients with clinical and pathological T stage, HPV status, positive lymph node number, and clinical outcome.

Analysis of the novel gene signature and its potential functions exhibited that KEGG was primarily enriched in primary immunodeficiency, intestinal immune network for IgA production, B cell receptor signaling pathway, and T cell receptor signaling pathway. Several immune pathways were observed in the low-risk group, indicating that the immune system was activated.

In terms of immune infiltration, the count of the effector memory CD8 T cells, activated B cells, activated CD8 T cells, follicular helper CD8 T cells, activated CD4 T cells, and natural killer cells were significantly different between the high- and low-risk groups. Interestingly, we also noted that the patients in the high-risk group displayed higher TMB and MSI scores. These findings suggested that the infiltration of a few specific immune cell subtypes could considerably affect the prognosis of HNSCC patients.

In HNSC, immune checkpoint blockade therapeutic strategies are of great clinical significance [12]. The negative relationship between risk scores and immune checkpoints, like TIGHT, PDCD1, CTLA4, LAG3, and IDO1, showed that the patients having low-risk scores could have a better immune microenvironment, which made them more likely to respond to ICIs. We also noted that the low-risk group responded to immunotherapy significantly better than the high-risk group, which led to the conclusion that patients with low risk were most likely to benefit from immunotherapy.

LncRNA plays an important regulatory role in several tumor types [33–36]. Most of the lncRNAs have a close association with the genes coded in the vicinity of certain mRNAs, and the interactions between the lncRNA-mRNA pairs [37, 38]. Extensive research has been done on the potential role of lncRNAs as novel biomarkers. Based on the current situation where only a handful of lncRNAs have been reported for HNSCC, our findings will offer a new approach to developing lncRNAs-related therapies for HNSCC. In our study, 109 cuproptosis-related lncRNAs were identified for constructing the lncRNA signature. 11 hub lncRNAs were selected for designing the model equation for risk assessment. This model was calibrated and validated using internal validation data. We noted that the cuproptosis-related lncRNA risk model was an effective and independent prognostic factor and could be used as an early marker for predicting the onset and progression of HNSCC with a good prediction performance. Additionally, we determined a significant correlation between the clinical-pathological features and risk signature. Furthermore, the lncRNA risk score showed a higher correlation with the tumor cells such as the CD4 memory-activated T cells, CD8 T cells, and naive B cells, which indicated an active anti-tumor immune response.

Cuproptosis, a novel and cryptic cell death model, presents a new therapeutic approach for cancer treatment. However, very few researchers have studied the relationship between cuproptosis and other cell death models [39]. In this study, we have integrated the cuproptosis biomarkers for predicting the treatment outcomes in HNSCC patients as well as identifying the potential therapeutic targets. The risk model we built is a novel cuproptosis-related biomarker for HNSCC prognosis and screening. The risk model demonstrates good prognostic predictive power. It can help clinical doctors to differentiate between high and low risk HNSCC patients to help individualize treatment. Additionally, it also showed tight correlation with TIICs, TME, immune function, immune checkpoints, and immunotherapy as the possible immune responses. The drawbacks of this study include that our prognostic model was both constructed and validated with retrospective data from public databases. A small sample size was used that limited its statistical significance. In addition, the results were not clinically verified. More prospective real-world data should be warranted to verify its clinical utility. Generally, we have identified novel cuproptosis-related biomarkers for HNSCC prognosis. These results could offer insights that could help in developing accurate and robust cancer therapy strategies.

## Figures and Tables

**Table 1:** Baseline information of TCGA.

TCGA (*N* = 501)		
Age (%)	19-39	18 (3.6)
	40-49	58 (11.6)
	50-59	145 (28.9)
	60-90	280 (55.9)
Gender (%)	Female	134 (26.7)
	Male	367 (73.3)
Stage (%)	Stage I-II	113 (22.6)
	Stage III-IV	374 (74.7)
	NA	14 (3.7)

**Table 2:** 10 cuproptosis-related genes.

FDX1 LIAS LIPT1 DLD DLAT PDHA1 PDHB MTF1 GLS CDKN2A

**Table 3:** 18 inflammatory genes in T cell-inflamed gene expression profile (GEP).

CCL5 CD27 CD274 CD276 CD8A CMKLR1 CXCL9 CXCR6 HLA-DQA1 HLA-DRB1 HLA-E IDO1 LAG3 NKG7 PDCD1LG2 PSMB10 STAT1 TIGIT

**Table 4:** The seven steps of cancer immunity cycle.

The seven steps of cancer immunity cycle	
Step 1	Release of cancer cell antigens
Step 2	Cancer antigen presentation
Step 3	Priming and activation
Step 4	Trafficking of immune cells to tumors
Step 5	Infiltration of immune cells into tumors
Step 6	Recognition of cancer cells by T cells
Step 7	Killing of cancer cells

## Data Availability

The public dataset used in this study is freely available at https://xenabrowser.net/, https://www.ncbi.nlm.nih.gov/, and https://pdc.cancer.gov/pdc/browse.

## Supplementary Materials

Supplementary Materials Figure S1. Survival analysis of CRG risk model. (a), (b) Survival analysis showed the prognosis of high-risk and low-risk patients. PFS: progression-free-survival; DSS: disease specific survival. (c)–(e) Correlation analysis of risk score and clinical characteristics. (f) Distribution of HNSCC patients based on the risk score. Risk curve and scatter plot for the risk score and survival status of each HNSCC case. The red and green dots represent death and survival, respectively. Heat map showing the expression profiles of cuproptosis-associated seven-genes in the high-risk group and the low-risk group. Figure S2. The IC50 of common chemotherapy agents between gene-related low- and high-risk groups. The IC50 of doxorubicin (a) and docetaxel (b) in the low-risk group were higher than those in the high-risk group. Figure S3. Evaluation of infiltrating immune cells and distribution of HNSCC patients based on the risk scores. (a)–(e) Box plot presents the differentially naive B cells (a), CD4 memory resting T cells (b), follicular helper T cells (c), memory activated CD4 T cells (d), and Tregs (e) between cluster 1 and cluster 2. (f) Differences in stromal score between cluster 1 and cluster 2. (g)–(h) Distribution of HNSCC patients based on the risk score in the train group (g) and the test group (h). Figure S4. Prognostic value of cuproptosis-associated lncRNA signature. The Kaplan-Meier curve showed that patients in different groups ((a) male patients; (b) patients with M0; (c)–(d) patients with different grade; (e)–(f) patients with different N; (g)–(h) patients with different stage) with high risk displayed a shorter overall survival than those with low risk. Figure S5. Prognostic value and correlation of the risk score with immune cells. (a), (b) Correlation analysis of lncRNA-related risk score and clinical characteristics. (c) lncRNA-related risk score was positively correlated with CD4 memory resting T cells.
