# Supplementary material for: The Prognostic Role of Cuproptosis in Head and Neck Squamous Cell Carcinoma Patients: A Comprehensive Analysis
Source: Dis Markers. 2022 Sep 2;2022:9996946. doi: 10.1155/2022/9996946 (PMC9463014; doi:10.1155/2022/9996946)
Supplement: Supplementary Materials — Figure S1. Survival analysis of CRG risk model. (a), (b) Survival analysis showed the prognosis of high-risk and low-risk patients. PFS: progression-free-survival; DSS: disease specific survival. (c)–(e) Correlation analysis of risk score and clinical characteristics. (f) Distribution of HNSCC patients based on the risk score. Risk curve and scatter plot for the risk score and survival status of each HNSCC case. The red and green dots represent death and survival, respectively. Heat map showing the expression profiles of cuproptosis-associated seven-genes in the high-risk group and the low-risk group. Figure S2. The IC50 of common chemotherapy agents between gene-related low- and high-risk groups. The IC50 of doxorubicin (a) and docetaxel (b) in the low-risk group were higher than those in the high-risk group. Figure S3. Evaluation of infiltrating immune cells and distribution of HNSCC patients based on the risk scores. (a)–(e) Box plot presents the differentially naive B cells (a), CD4 memory resting T cells (b), follicular helper T cells (c), memory activated CD4 T cells (d), and Tregs (e) between cluster 1 and cluster 2. (f) Differences in stromal score between cluster 1 and cluster 2. (g)–(h) Distribution of HNSCC patients based on the risk score in the train group (g) and the test group (h). Figure S4. Prognostic value of cuproptosis-associated lncRNA signature. The Kaplan-Meier curve showed that patients in different groups ((a) male patients; (b) patients with M0; (c)–(d) patients with different grade; (e)–(f) patients with different N; (g)–(h) patients with different stage) with high risk displayed a shorter overall survival than those with low risk. Figure S5. Prognostic value and correlation of the risk score with immune cells. (a), (b) Correlation analysis of lncRNA-related risk score and clinical characteristics. (c) lncRNA-related risk score was positively correlated with CD4 memory resting T cells. [file 9996946.f1.docx]

**Supplemental Files**


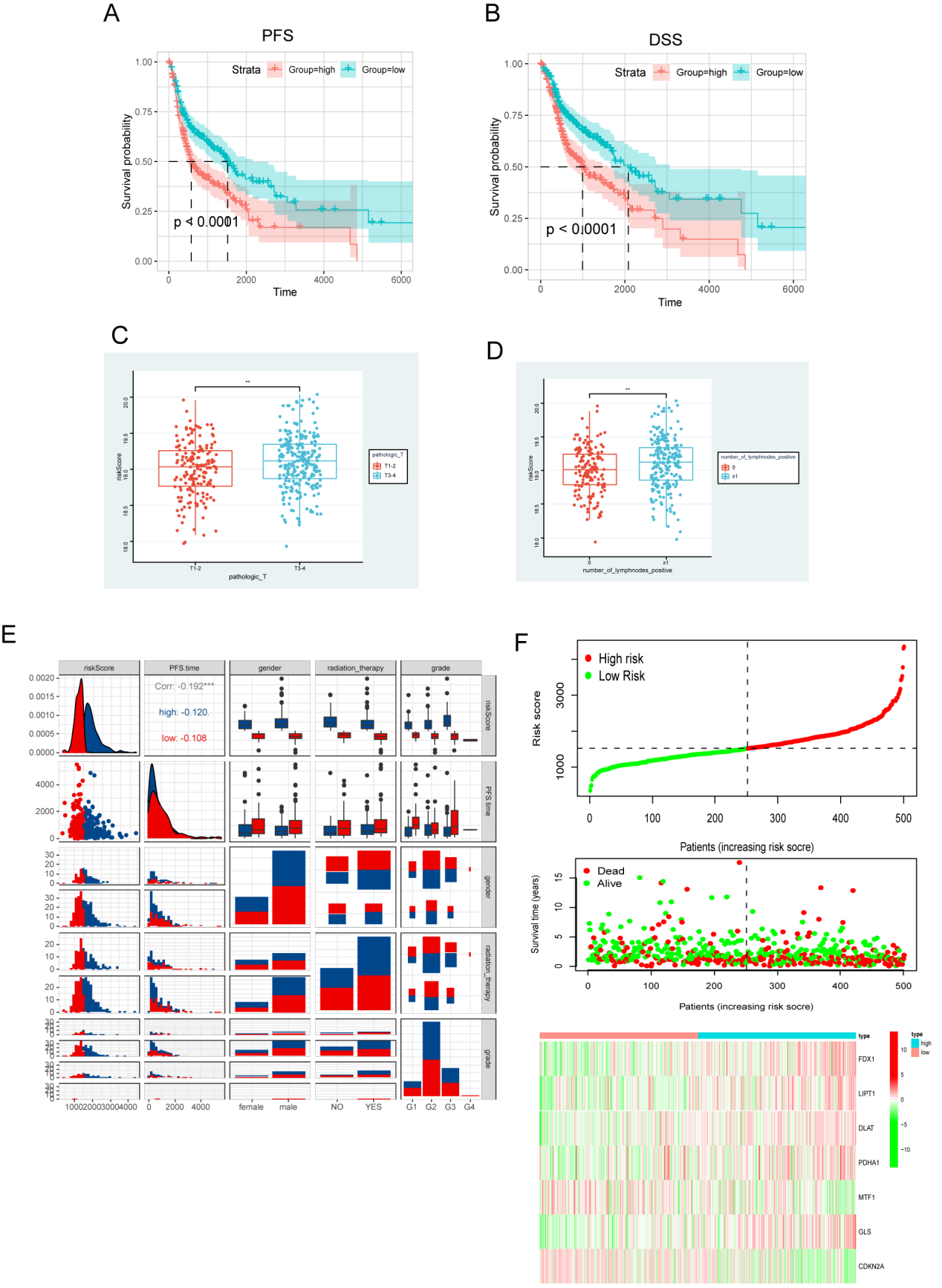


**Fig. S1.** **survival analysis of CRG risk model.** (A-B)Survival analysis showed the prognosis of high-risk and low-risk patients. PFS: Progression-Free-Survival; DSS: Disease Specific Survival. (C-E) Correlation analysis of risk score and clinical characteristics. (F) Distribution of HNSCC patients based on the risk score. Risk curve and scatter plot for the risk score and survival status

of each HNSCC case. The red and green dots represent death and survival, respectively. Heat map showing the expression profiles of cuproptosis-associated seven-genes in the high-risk group and the low-risk group.


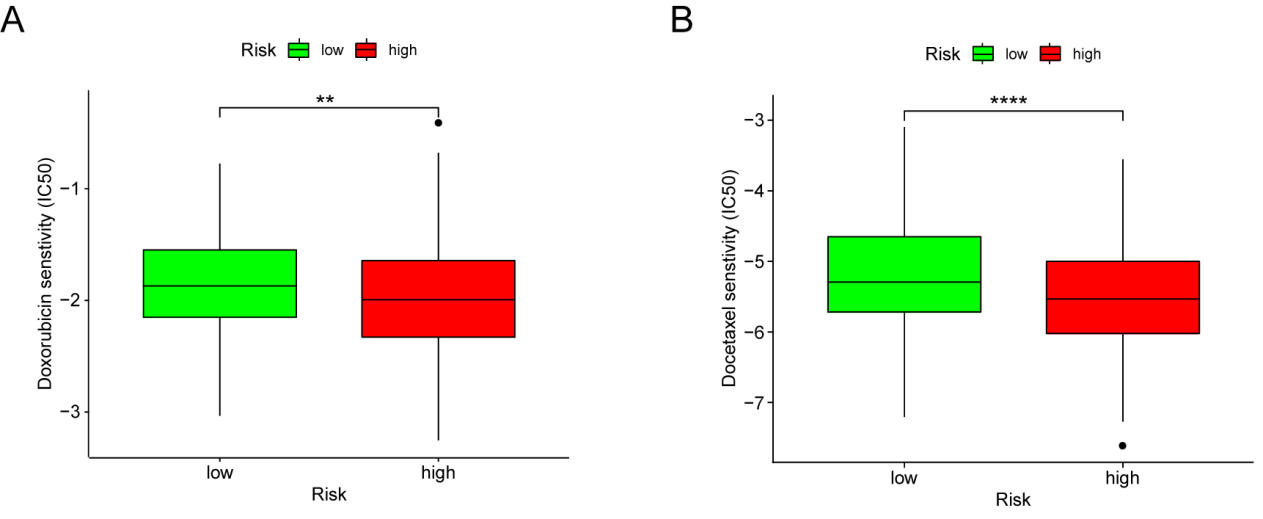


**Fig. S2. The IC50 of common chemotherapy agents between gene-related low- and high-risk groups.** The IC50 of doxorubicin (A) and docetaxel (B) in the low-risk group were higher than those in the high-risk group.


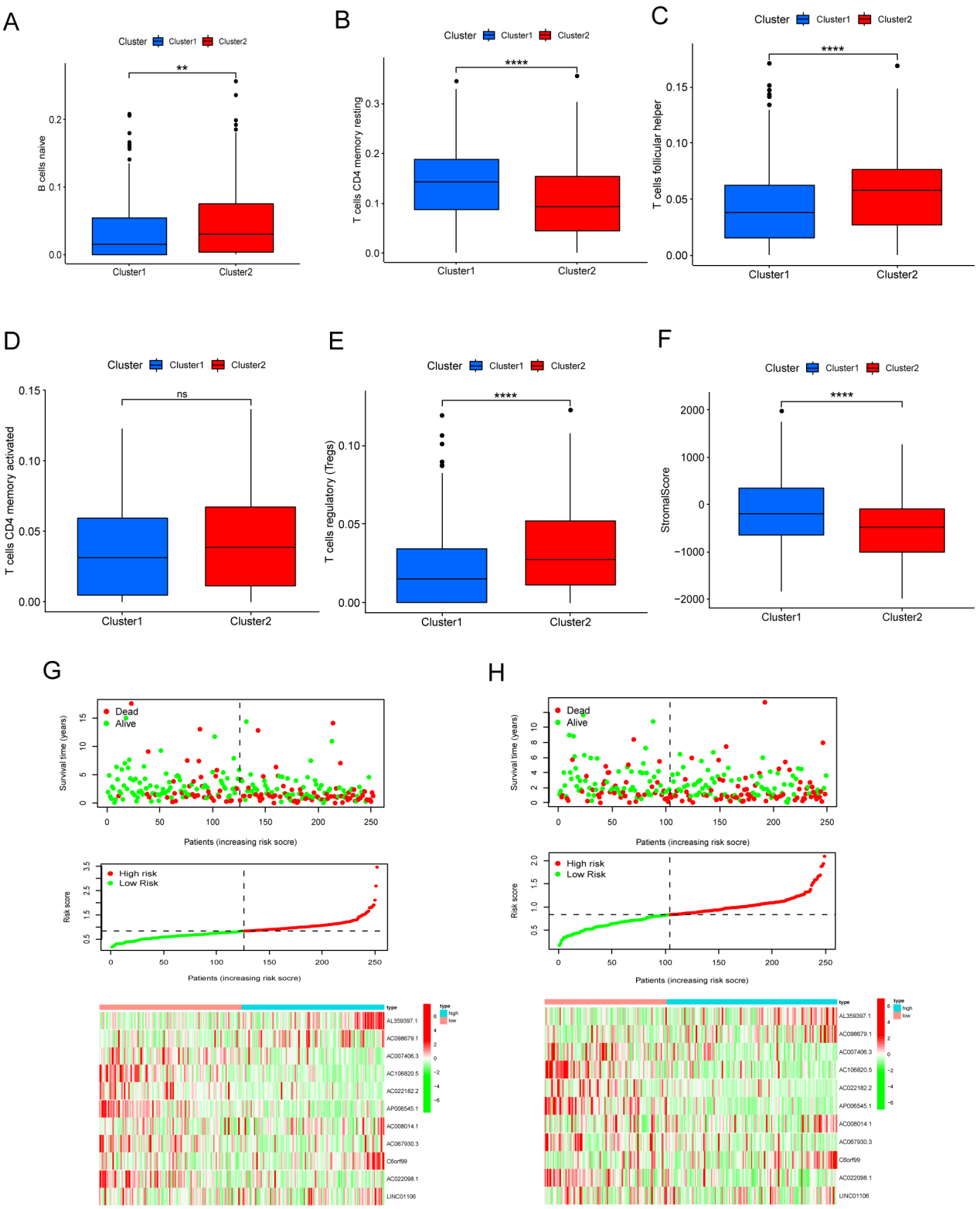


**Fig. S3. Evaluation of infiltrating immune cells and distribution of HNSCC patients based on the risk scores.** (A-E)Box plot presents the differentially naive B cells (A), CD4 memory resting T cells (B), follicular helper T cells (C), memory activated CD4 T cells (D), and Tregs (E) between cluster 1 and cluster 2. (F)Differences in stromal score between cluster 1 and cluster 2. (G-H)Distribution of HNSCC patients based on the risk score in the train group (G) and the test group (H).


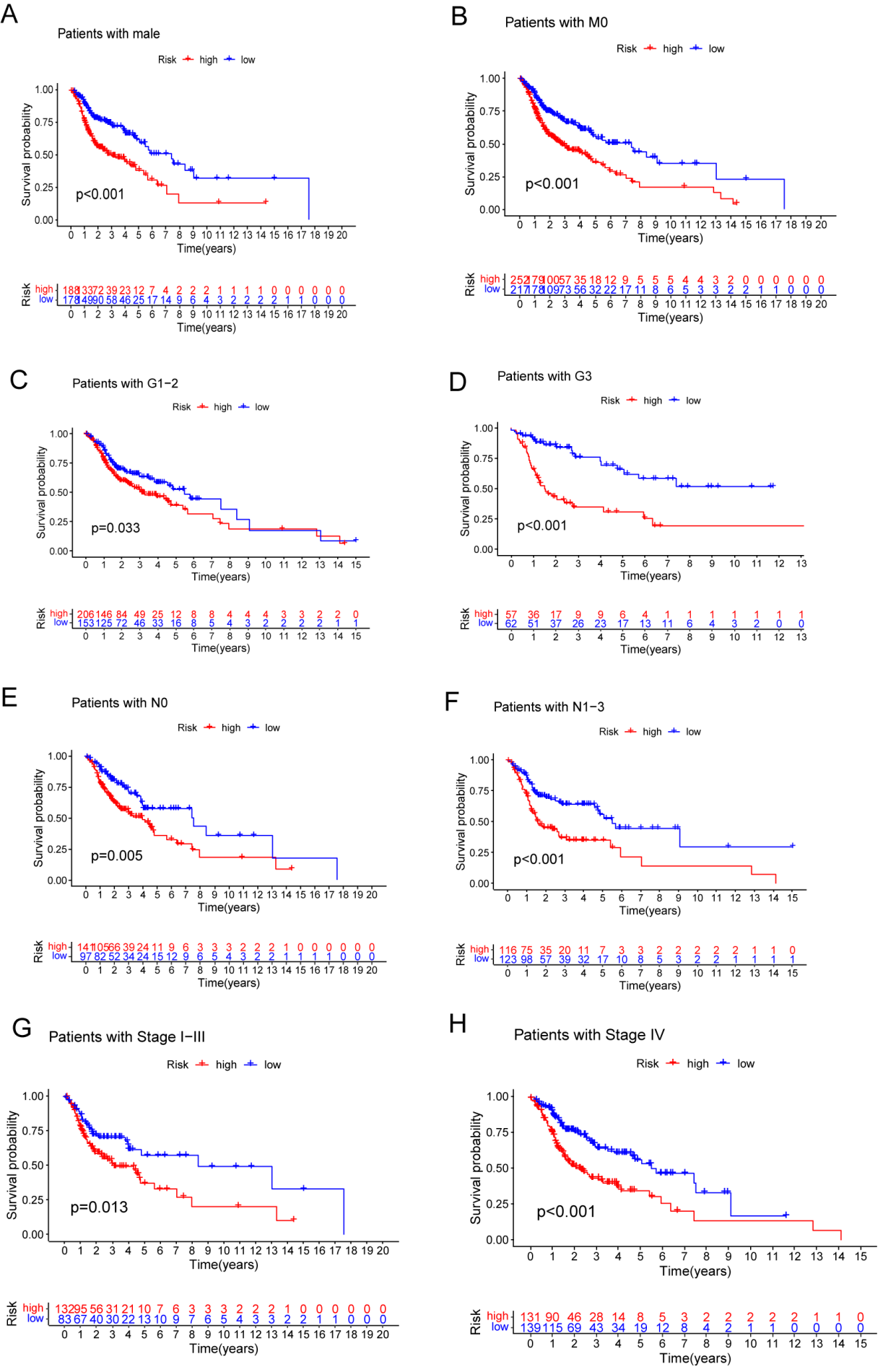


**Fig. S4. Prognostic value of cuproptosis-associated lncRNA signature.** The Kaplan-Meier curve showed that patients in different groups (A: male patients; B: patients with M0; C-D: patients with different grade; E-F: patients with different N; G-H: patients with different stage) with high risk displayed a shorter overall survival than those with low risk.


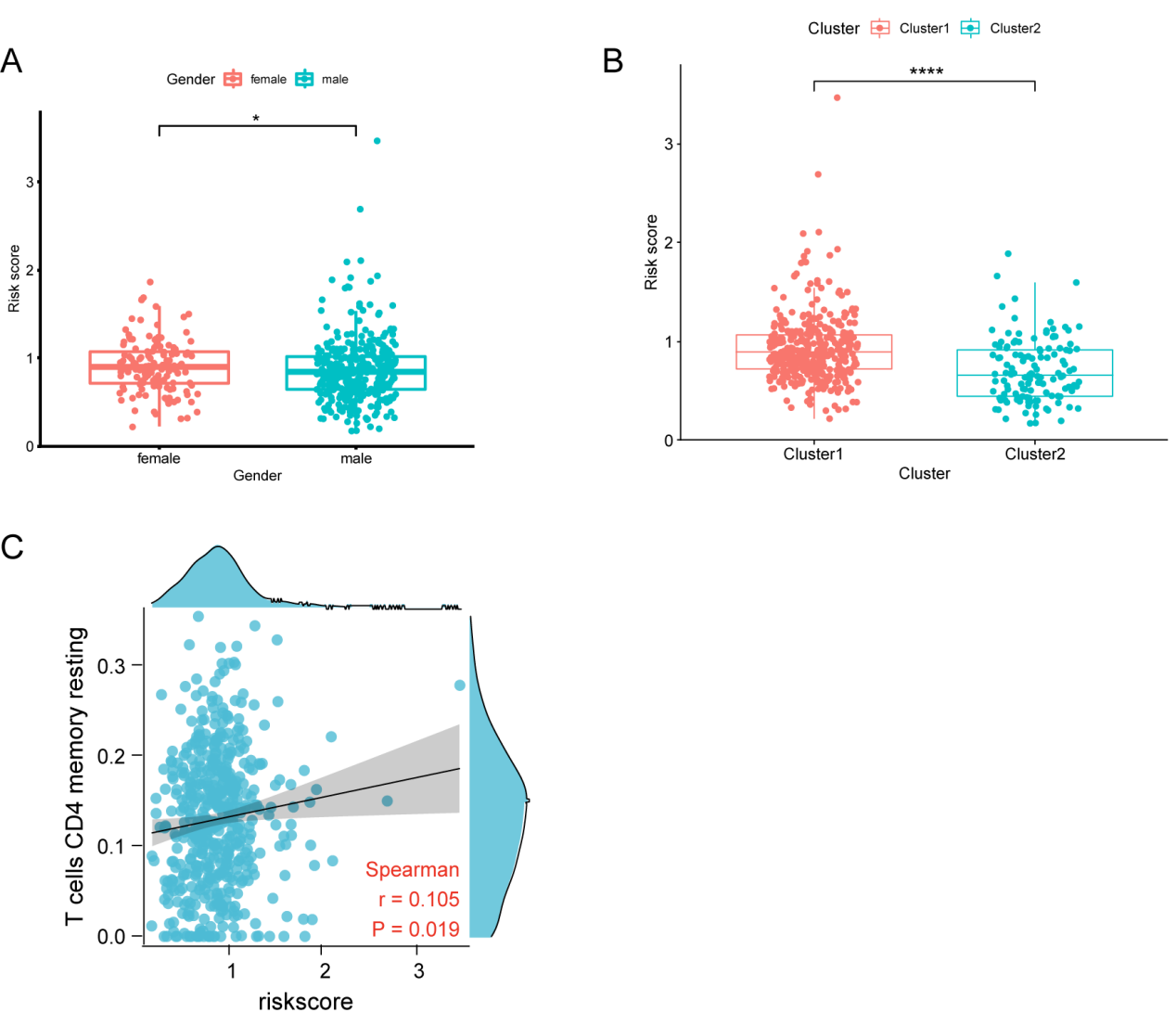


**Fig. S5. Prognostic value and correlation of the risk score with immune cells.** (A-B) Correlation analysis of lncRNA-related risk score and clinical characteristics. (C) lncRNA-related risk score was positively correlated with CD4 memory resting T cells.
